# Monitoring the elimination of *gambiense* human African trypanosomiasis in the historical focus of Batié, South–West Burkina Faso

**DOI:** 10.1051/parasite/2022024

**Published:** 2022-05-11

**Authors:** Charlie Franck Alfred Compaoré, Jacques Kaboré, Hamidou Ilboudo, Lian Francesca Thomas, Laura Cristina Falzon, Mohamed Bamba, Hassane Sakande, Minayégninrin Koné, Dramane Kaba, Clarisse Bougouma, Ilboudo Adama, Ouedraogo Amathe, Adrien Marie Gaston Belem, Eric Maurice Fèvre, Philippe Büscher, Veerle Lejon, Vincent Jamonneau

**Affiliations:** 1 Centre International de Recherche-Développement sur l’Élevage en zone Subhumide, Unité de recherche sur les maladies à vecteurs et biodiversité 01 BP 454 Bobo-Dioulasso 01 Burkina Faso; 2 Université Nazi Boni, Unité de Formation et de Recherche Sciences et Techniques 01 BP 1091 Bobo-Dioulasso Burkina-Faso; 3 Institut de Recherche en Sciences de la Santé, Unité de Recherche Clinique de Nanoro 11 BP 218 Ouagadougou CMS 11 Burkina Faso; 4 International Livestock Research Institute PO Box 30709 Nairobi 00100 Kenya; 5 University of Liverpool, Institute of Infection, Veterinary and Ecological Sciences Liverpool L69 3BX United Kingdom; 6 Institut Pierre Richet, Unité de Recherche et de Formation Trypanosomoses et Leishmanioses 01 BP 1500 Bouake Côte d’Ivoire; 7 Programme National de Lutte contre les Maladies Tropicales Négligées 03 BP 7009 Ouagadougou 03 Burkina Faso; 8 Institute of Tropical Medicine, Department of Biomedical Sciences Nationalestraat 155 2000 Antwerp Belgium; 9 Institut de Recherche pour le Développement, UMR INTERTRYP IRD-CIRAD, Université de Montpellier, TA A-17/G, Campus International de Baillarguet 34398 Montpellier France

**Keywords:** Human African trypanosomiasis, *Trypanosoma brucei gambiense*, Elimination, Diagnosis, Rapid diagnostic test, Specificity, Dried blood spot, Burkina Faso

## Abstract

The World Health Organisation has targeted the elimination of human African trypanosomiasis (HAT) as zero transmission by 2030. Continued surveillance needs to be in place for early detection of re-emergent cases. In this context, the performance of diagnostic tests and testing algorithms for detection of the re-emergence of *Trypanosoma brucei gambiense* HAT remains to be assessed. We carried out a door-to-door active medical survey for HAT in the historical focus of Batié, South–West Burkina Faso. Screening was done using three rapid diagnostic tests (RDTs). Two laboratory tests (ELISA/*T. b. gambiense* and immune trypanolysis) and parasitological examination were performed on RDT positives only. In total, 5883 participants were screened, among which 842 (14%) tested positive in at least one RDT. Blood from 519 RDT positives was examined microscopically but no trypanosomes were observed. The HAT Sero-*K*-Set test showed the lowest specificity of 89%, while the specificities of SD Bioline HAT and rHAT Sero-Strip were 92% and 99%, respectively. The specificity of ELISA/*T. b. gambiense* and trypanolysis was 99% (98–99%) and 100% (99–100%), respectively. Our results suggest that *T. b. gambiense* is no longer circulating in the study area and that zero transmission has probably been attained. While a least cost analysis is still required, our study showed that RDT preselection followed by trypanolysis may be a useful strategy for post-elimination surveillance in Burkina Faso.

## Abstract (fr)

L’Organisation mondiale de la santé a ciblé l’élimination de la trypanosomiase humaine africaine (THA) comme transmission zéro d’ici 2030. Une surveillance continue doit être mise en place pour la détection précoce des cas réémergents. Dans ce contexte, la performance des tests de diagnostic et des algorithmes de test pour la détection de la réémergence de la THA de *Trypanosoma brucei gambiense* reste à évaluer. Nous avons réalisé une enquête médicale en porte-à-porte pour la THA dans le foyer historique de Batié, au sud-ouest du Burkina Faso. Le dépistage a été effectué à l’aide de trois tests de diagnostic rapide (TDR). Deux tests de laboratoire (ELISA/*T. b. gambiense* et trypanolyse immunitaire) et un examen parasitologique ont été effectués uniquement sur les TDR positifs. Au total, 5883 participants ont été dépistés, parmi lesquels 842 (14 %) ont été testés positifs dans au moins un TDR. Le sang de 519 TDR positifs a été examiné au microscope mais aucun trypanosome n’a été observé. Le test HAT Sero-K-Set a montré la spécificité la plus faible de 89 %, tandis que les spécificités de SD Bioline HAT et rHAT Sero-Strip étaient de 92 % et 99 %, respectivement. La spécificité d’ELISA/*T. b. gambiense* et de la trypanolyse étaient respectivement de 99 % (98–99 %) et 100 % (99–100 %). Nos résultats suggèrent que *T. b. gambiense* ne circule plus dans la zone d’étude et que la transmission zéro a probablement été atteinte. Bien qu’une analyse de moindre coût soit toujours nécessaire, notre étude a montré qu’une présélection par TDR suivie d’une trypanolyse peut être une stratégie utile pour la surveillance post-élimination au Burkina Faso.

## Introduction

Human African trypanosomiasis (HAT), commonly referred to as sleeping sickness, is an infection caused by *Trypanosoma brucei* parasite subspecies that are transmitted by the bite of a fly of the genus *Glossina* (tsetse fly). In western and central Africa, *Trypanosoma brucei* (*T. b.*) *gambiense* is responsible for the chronic form of HAT, while *T. b. rhodesiense* is responsible for the acute form in East Africa [5].

Sleeping sickness epidemics have caused devastation in Africa in the past, with millions of victims [18]. The last epidemic occurred towards the end of the 20th century. Around that time, Burkina Faso reported between 10 and 20 *gambiense* HAT cases annually [44], subsequently attributed to Côte d’Ivoire as the country of infection [40]. Since 1993, no native Burkinabe HAT cases have been reported, except for one case in 2015 detected following implementation of a passive surveillance system [14]. As a result, Burkina Faso is encouraged to request validation of elimination of HAT as a public health problem [18], while the country progresses towards the WHO 2030 target of interruption of transmission (zero cases) [5].

However, presence of tsetse flies, residual cases, asymptomatic carriers, imported cases, and animal reservoirs may provoke re-emergence of *gambiense* HAT [3, 4, 19], as has been observed in the recent past [39, 43]. These factors represent a threat for the elimination process in historical foci of HAT, such as South–West Burkina Faso [9, 14]. Efficient post-elimination monitoring is required to ensure sustainability of elimination [18].

In places where coverage using active mass screening and/or passive surveillance remains low, dried blood spots (DBS) can easily be collected by non-specialist health workers and sent to a reference laboratory for testing [38]. Nonetheless, the costs of testing large numbers of DBS can be high, depending on the test and testing algorithm chosen. A cheap and simple screening process, reducing the number of DBS to be tested in the reference laboratory, has the potential to reduce costs. The combination of door-to-door visits by a mobile team carrying out rapid diagnostic tests (RDTs) for HAT [41], and sampling blood on filter paper for subsequent reference laboratory tests based on RDT results, is considered a feasible active case finding strategy [20]. In this context, the diagnostic performance of RDTs, of high throughput laboratory tests, and of diagnostic algorithms for HAT elimination monitoring and for early detection of *gambiense* HAT re-emergence remains to be assessed. We therefore conducted a door-to-door medical survey using three different RDTs and collection of DBSs in the historical focus of Batié in South–West Burkina Faso [9].

## Material and methods

### Ethics

All investigations were conducted in accordance with the Declaration of Helsinki. The activities were part of the Burkinabe arm of the DiTECT-HAT-WP3 multi-country diagnostic clinical trial (Diagnostic Tools for Human African Trypanosomiasis Elimination and Clinical Trials, work package 3, post-elimination monitoring). Ethical approval was obtained from the Advisory Committee on Deontology and Ethics (plenary meeting of 17–20 October 2016) of the French National Institute for Research on Sustainable Development (IRD), from the Institutional Review Board of the Institute of Tropical Medicine in Antwerp Belgium (reference 1134/16), from the Ethics Committee of the University of Antwerp (Belgian registration number B300201730929), and from the National Ethics Committee of the Ministry of Health of Burkina Faso (deliberation number 2017-5-063). The DiTECT-HAT-WP3 project is registered on https://www.ClinicalTrials.gov, ID NCT04099628. Before inclusion in the study, each potential study participant was informed about the study in her/his language. Written informed consent was obtained from all study participants. For minors, legal representatives provided written informed consent and, in addition, assent was obtained from the participating minor.

### Study area, inclusion and sampling procedures

**Figure 1:**
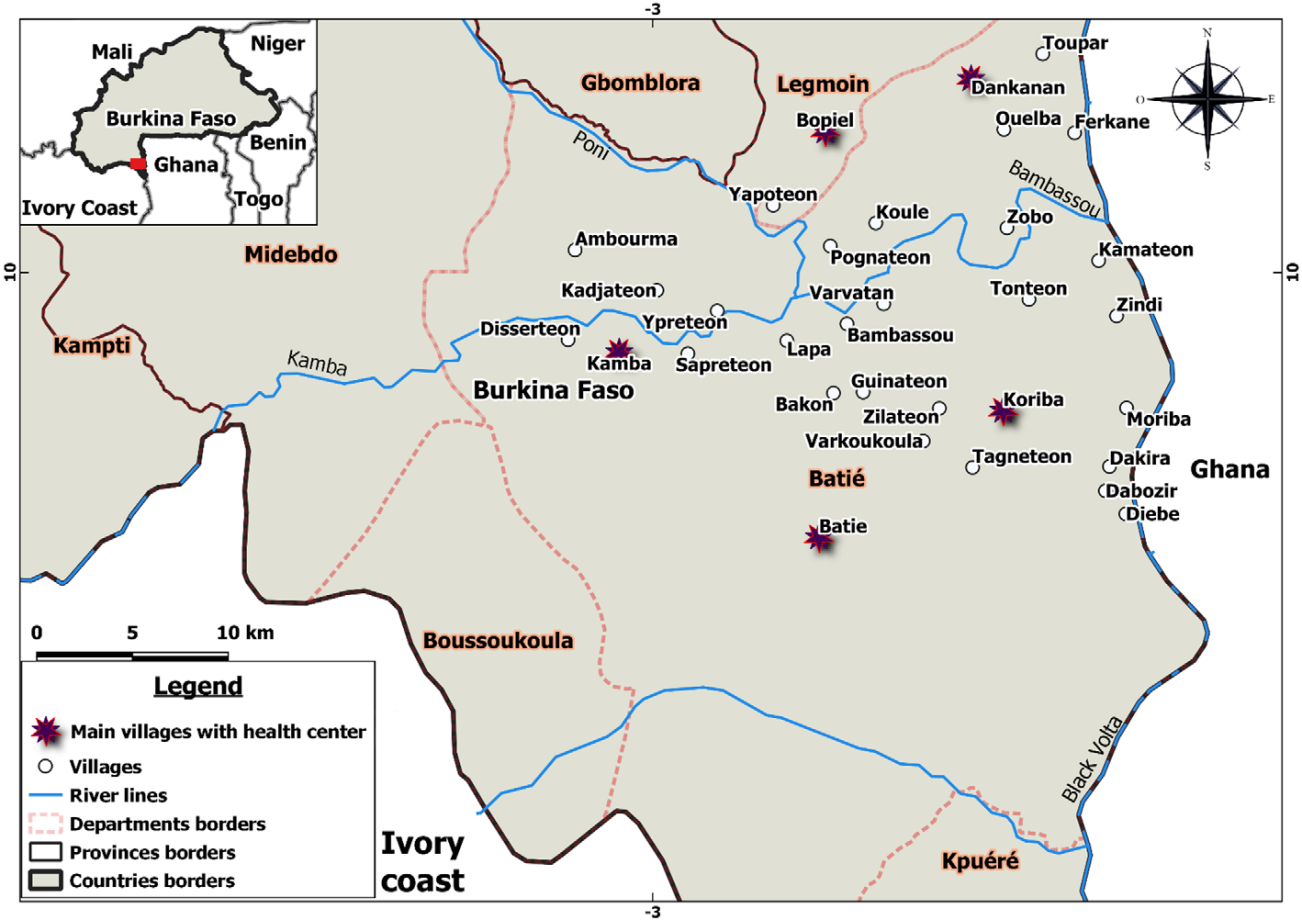
Study area indicating the 29 villages and the five health centers in Batié health district within Noumbiel Province*.* Kamba and Koriba have a health center and are part of the 29 selected villages, while Bopiel, Batié and Dankanan have a health center but the village was not included.

Batié is a town in South–West Burkina Faso in the border area with Côte d’Ivoire (Fig. 1), and was particularly affected by HAT in the 1940s and 1950s with thousands of cases [16]. Thanks to control efforts, prevalence gradually decreased and no re-emergence has been reported since [9], despite the potential risk of reintroduction of the parasite due to important migratory flow between this area and Côte d’Ivoire HAT foci [10].

Activities took place between October 2019 and February 2020 in Batié Health District (Noumbiel Province) that had a population of 98,883 inhabitants in 2019 [23]. Batié health district is composed of five departments (Batié, Boussoukoula, Kpuéré, Legmoin and Midebdo) (Fig. 1).

During a preparatory mission, 29 villages (28 in Batié Department and one in Legmoin Department) were selected. The selection criteria were accessibility by car or motorcycle and a population of fewer than 1000 inhabitants. Each of these villages is attached to a health center (HC) and belongs to that HC area (HCA): Baupiel in Legmoin Department, and Kamba, Koriba, Batié and Dankanan in Batié Department. The local health authorities, the health staff of the five HC, as well as the customary authorities of the 29 villages, were informed about HAT and the study.

Next, a door-to-door medical survey was carried out in each village where houses are grouped in compounds. All permanent residents (resident for a minimum of one year) were eligible for the study. Individuals meeting one of the following criteria were excluded: persons who had previously been treated for HAT (irrespective of time elapsed since treatment), and children under 4 years of age.

In each compound, a community-based health worker explained the study in the local language and obtained written informed consent and, for minors, assent. Next, a nurse collected approximately 1 mL of venous blood using a vacutainer^®^ heparinized tube. The blood specimen was used for carrying out the three RDTs (see below), and for preparing the DBS on Whatman^®^ Grade 4 qualitative filter paper (110 mm diameter). Filter paper was mounted on a stick and 16 drops of 30 μL heparinized blood were applied using a micropipette. The DBS were left to dry for at least 1 h. When the DBS had dried (indicated by the dark brown color), each filter paper was inserted into an individual envelope for transport to the reference laboratory. A maximum of 10 envelopes were inserted in an air-tight plastic zipper bag (Rajapack) in which about 30 g of dry silica gel (VWR) were added.

Simultaneously, the following data were recorded electronically for each participant: GPS coordinates of the residence, HCA, gender, age, RDTs results and corresponding pictures from the RDTs. The aim was to test about 6000 people.

### Rapid diagnostic test procedure

Three RDTs were performed simultaneously following the manufacturers’ instructions, except for the sampling of blood that was done by drawing blood from the heparinized tube with a micropipette. These tests were: SD Bioline HAT (Abbott Diagnostics, South Korea), HAT Sero-*K*-Set (Coris BioConcept, Belgium), and rHAT Sero-Strip (Coris BioConcept, Belgium).

### Laboratory tests

Trypanolysis was carried out for all participants who tested positive to at least one RDT. This antibody mediated complement lysis test was performed as previously described [6, 24], with minor modifications for DBS on Whatman^®^ Grade 4 paper. Trypanolysis was carried out with *T. b. gambiense* variable antigen type clones LiTat 1.3 and LiTat 1.5, which were manipulated in separate experiments. Briefly, trypanosomes were expanded in mice. Once parasitemia between antilog 8.1 and 8.4 trypanosomes per mL was reached according to the Matching Method [21], the trypanosomes were harvested. Blood was taken from the tail of the mouse, and dilutions were made in guinea pig serum to adjust the parasitemia of the suspension to 5–10 trypanosomes per field at ×400 magnification. In parallel, two discs were punched out of each DBS with a 6 mm perforator and deposited in one well of a microplate. As a positive and negative control, positive serum and blank filter paper were used, respectively. Then 40 μL of guinea pig serum were dispensed into each well and the plate was sealed and agitated for 10 s on a plate shaker (maximum speed), after which it was left to incubate for 60 min at ambient temperature on a plate shaker (minimum speed). After incubation, 10 μL of the trypanosome suspension were added to each well. The plate was agitated for 10 s (maximum speed) and incubated for 90 min at ambient temperature with 10 s agitation steps every 15 min. Then, 8 μL of the mixture were deposited on a microscope slide, covered with a cover slip (18 × 18 mm) and examined under a phase contrast microscope (×400). The trypanolysis test was considered positive when more than 50% of the trypanosomes were lyzed. The DBS was considered positive in trypanolysis when it was positive with at least one of the two variable antigen type clones. All personnel working with laboratory animals had a level 1 or 2 animal experimentation certificate. CIRDES (Centre International de recherche-développement sur l’élevage en zone subhumide) approved animal experimentation for trypanolysis within the framework of HAT diagnosis.

All participants who tested positive to at least one RDT were also tested in ELISA/*T. b. gambiense* using *T. b. gambiense* LiTat 1.3 and LiTat 1.5 variant surface glycoprotein (VSG) as antigens, as previously described [20]. One 6 mm DBS confetti (Whatman^®^ 4) was eluted in 720 μL PBS-Blotto-Tween overnight at 4 °C. Half of the ELISA/*T. b. gambiense* plate was coated with 150 μL of antigen (a mixture of LiTat.1.3 and 1.5 VSG diluted each to a concentration of 1 μg/mL in PBS), while the other half was left empty (antigen-free wells), and the plate was left overnight at 4 °C. Plates were saturated with PBS-Blotto and incubated for 1 h at ambient temperature, after which they were washed three times with PBS-Tween. Positive and negative control serum were diluted 1/150 in PBS-Blotto, and 150 μL of each control specimen were added to antigen-containing and antigen-free wells, in quadruple for the positive control and in duplicate for the negative control. A conjugate control (PBS-Blotto without serum) was also included in duplicate. Then, 150 μL of DBS eluate were added in duplicate to antigen-containing and antigen-free wells, and the plate was incubated for 30 min at ambient temperature. After three washes, 150 μL of peroxidase-conjugated AffiniPure Goat (Anti-Human IgG, Jackson ImmunoResearch, West Grove, PA, USA) diluted 1/40.000 in PBS-Tween were dispensed into each well of the microplate and incubated for 30 min. After five washes with PBS-Tween, the reaction was revealed by adding 150 μL of substrate/chromogen solution (ABTS) to each well and incubating for 1 h in the dark. The optical density (OD) was measured at 405 nm. The average OD of the duplicates was calculated, and the OD of antigen-free wells was subtracted from the corresponding antigen-containing wells. Reactivity of each specimen was expressed as percent positivity of the positive control serum. A DBS result was considered positive when its percentage positivity was higher than 30.

### Parasitological test

Participants who tested positive in at least one RDT were invited for parasitological examination. Participants who were also positive in ELISA/*T. b. gambiense* and/or trypanolysis were invited for an additional parasitological examination in case the first one was negative. Parasitological examinations were conducted 6 months after the RDT screening, with an interval of two weeks between the first and second parasitological examination when applicable. For parasitology, the mini anion-exchange/centrifugation technique (mAECT) was performed on 500 μL of venous blood collected on heparin, following the manufacturer’s instructions (INRB, Kinshasa, Democratic Republic of Congo).

### Statistical analysis

The data were imported from the tablets and cleaned in Microsoft Excel, version 2016. Analyses were performed using R Studio, version 1.2.1335 software using packages tidyverse and ggpubr for constructing pyramids. First, the Fisher Exact test was used to determine if there were unconditional associations between positivity to the individual RDTs and gender; and between overall RDT positivity and gender, health zone, and age. Next, we performed a multivariable logistic regression to determine whether age, HCA, and gender were conditionally associated with overall RDT positivity. A *p*-value <0.05 was considered statistically significant. For each RDT and laboratory test, and for parallel and series combinations of RDTs, we estimated the diagnostic specificity and corresponding confidence intervals using the online EpiTools package (https://epitools.ausvet.com.au/ciproportion).

## Results

### Description of the study population

**Figure 2:**
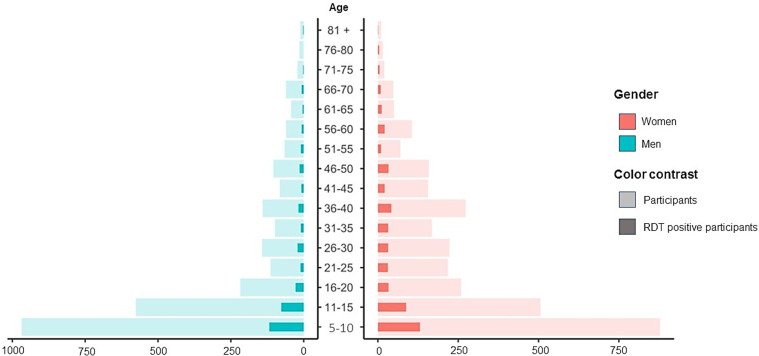
Population age pyramid representing the age and gender distribution of the study population, and the frequency of overall rapid diagnostic test positivity in each age and gender stratum.

In total, 5883 participants from the five HCA were included. Koriba was the most populated and most accessible HCA, and had the highest population adherence, resulting in 3175 inclusions (54.0%). In the HCA of Batié, 599 (10.2%) individuals were included, in Baupiel 628 (10.7%), in Dankanan 662 (11.3%), and in Kamba 819 (13.9%). The mean age of the study participants was 24 years (range = 5–110), and the women to men ratio was 1.15:1 (3158/2725) (Fig. 2).

### Diagnostic test results

**Figure 3:**
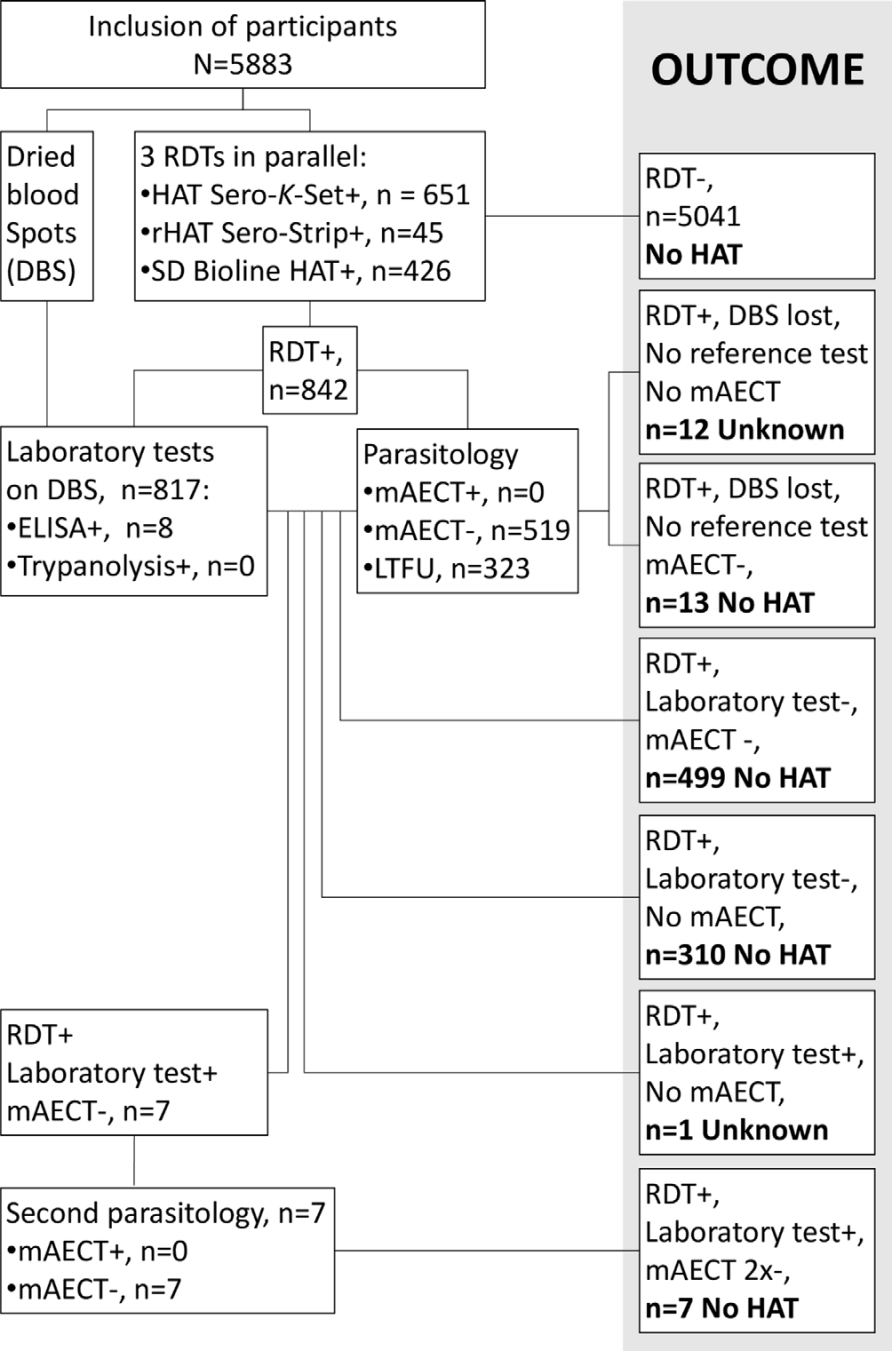
Flow chart of activities, test results and diagnostic outcome of participants. RDT = Rapid Diagnostic Test; DBS = dried blood spot; mAECT = mini anion-exchange/centrifugation technique; LTFU = lost to follow-up.

A flow chart of the activities, including the results of the RDTs, laboratory tests (ELISA/*T. b. gambiense* and trypanolysis) and parasitological tests, is shown in Figure 3. The overall seroprevalence among the study participants, based on positivity in at least one RDT, was 14.3% (842/5883, CI: 13.4–15.2%). Seroprevalence based on HAT-Sero-*K*-Set was 11.1% (651/5883, CI: 10.3–11.9), 7.2% on SD Bioline HAT (426/5883, CI: 6.6–7.9%) and 0.8% on rHAT Sero-Strip (45/5883, CI: 0.6–1.0%).

**Figure 4:**
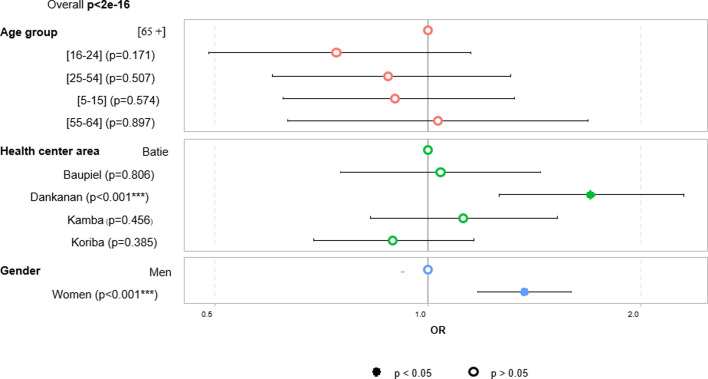
Multivariable logistic regression showing the association between overall rapid diagnostic test positivity and age group, health center area, and gender.

There was a significant difference between the number of women (16.0%, 504/3158, CI: 14.7–17.3%) and men (12.1%, 331/2725, CI: 11.0–13.4%) who tested positive to at least one RDT (*p* < 0.001) (Fig. 2). Women were significantly more commonly positive than men for both HAT Sero-*K*-Set (60.4%, 393/651, CI: 56.6–64.0%, *p* < 0.001) and SD Bioline HAT (62.7%, 267/426, CI: 58.0–67.1%, *p* < 0.001), while no significant difference between men and women was observed for rHAT Sero-Strip. When comparing the five HCAs, the proportion of seropositives on at least one RDT was significantly higher in Dankanan, compared to the other areas (21.6%, 143/662, CI: 18.6–25.0%, *p* < 0.001). There was no significant difference between the age groups. Based on the multivariable logistic regression (Fig. 4), the odds for being positive on at least one RDT were 1.37 (CI: 1.18–1.59%) higher for women (compared to men), and 1.70 (CI: 1.26–2.30%) when living in Dankanan HCA (compared to the reference Batié HCA).

Results of the laboratory tests were available for 817 of the 842 RDT-positive individuals. The volume of blood collected for the remaining 25 was insufficient to prepare a DBS. Among these 817, 8 were positive in ELISA/*T. b. gambiense*. None tested positive in trypanolysis. The ELISA/*T. b. gambiense* seroprevalence among the RDT positives was 0.98% (8/817, CI: 0.50–1.92%). Results of the eight ELISA/*T. b. gambiense* positive individuals are detailed in Table 1. Six were women, five were positive to the HAT Sero-*K*-Set or the SD Bioline HAT (two were positive to both of these RDT tests). Only one ELISA/*T. b. gambiense* positive participant was only positive to the rHAT Sero-Strip.

**Table 1:** Detailed serological and parasitological test results of 8 rapid diagnostic test positives with positive ELISA/*T. b. gambiense*. W: women; M: men.

Gender/age	HAT Sero-*K*-Set	rHAT Sero-Strip	SD Bioline HAT	Health center area	Village
W/11	+	−	+	Koriba	Diebe
M/12	+	−	+	Koriba	Zindi
W/14	−	+	−	Koriba	Zilateon
W/17	−	−	+	Koriba	Koriba
W/50	+	−	+	Koriba	Tagneteon
M/58	−	−	+	Koriba	Dakira
W/60	+	−	−	Batié	Varvatan
W/70	+	−	−	Koriba	Koriba

As shown in Figure 3, 519 of the 842 identified RDT positives (61.6%), including 7 of the 8 ELISA/*T. b. gambiense* positives, could be traced back for parasitological testing using mAECT but no trypanosomes were identified. The seven RDT and ELISA/*T. b. gambiense* positives re-tested negative with mAECT two weeks later. The prevalence of parasitologically confirmed HAT in the study area was therefore 0% (0/519, 95% CI: 0–0.0073%).

### Diagnostic specificity of the tests

**Figure 5:**
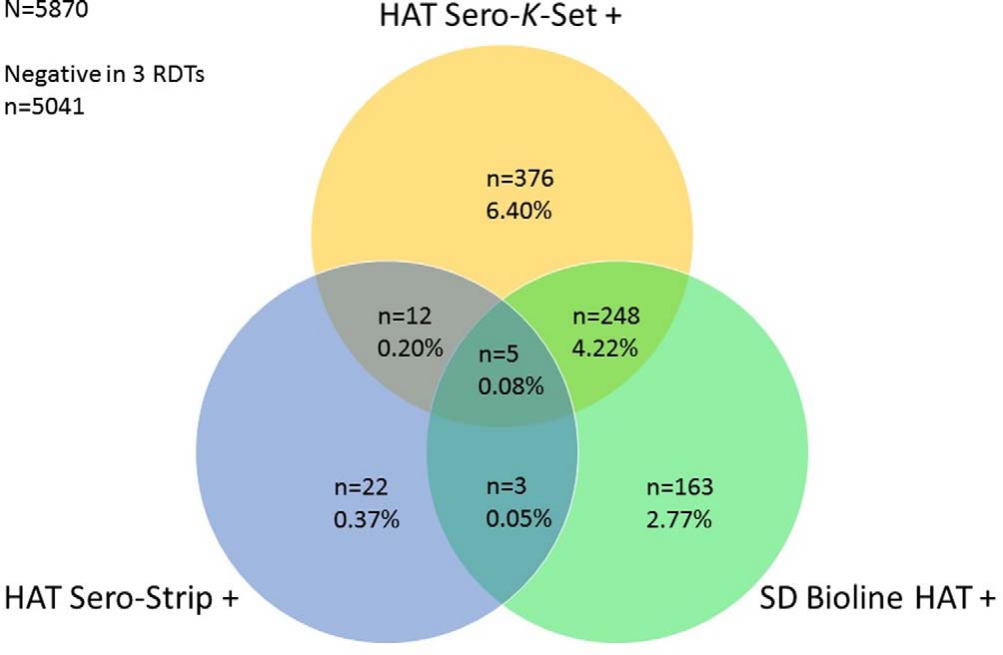
Venn diagram presenting the results of the 829 study participants who tested positive to at least one of the three rapid diagnostic tests for HAT.

Individuals were considered free of HAT (Fig. 3) if (1) they were negative to the three RDTs (*n* = 5041); (2) they tested positive in an RDT, did not undergo laboratory tests and were negative in mAECT (*n* = 13); (3) they tested positive in one RDT but were negative in ELISA/*T. b. gambiense* and trypanolysis, and the mAECT was either negative (*n* = 499) or not performed (*n* = 310); or (4) they tested positive in one RDT, were positive in ELISA/*T. b. gambiense*, negative in trypanolysis and twice negative in mAECT (*n* = 7). The status of RDT positives who did not undergo other tests and were lost to follow-up (*n* = 12) and the RDT positive who tested ELISA/*T. b. gambiense* positive but on whom no mAECT could be performed (*n* = 1), were considered unknown. RDT specificity was therefore estimated considering only people who were HAT free (5041 RDT negative and 829 RDT positive, N = 5870) (Table 2). Detailed results of the 829 RDT positive individuals are summarized in the Venn diagram (Fig. 5). Only 5 (0.08%) participants were positive to all three RDTs, with the majority (561/829, 67.7%) of participants only testing positive in one of the three RDTs.

**Table 2:** Specificity of individual HAT diagnostic tests and test combinations, performed in parallel or in series.

Diagnostic test	Total positive	% specificity (95% CI)	test negative/HAT free^a^
rHAT Sero-Strip	42	99.3 (99.0–99.5)	5828/5870
SD-Bioline HAT	419	92.9 (92.2–93.5)	5451/5870
HAT Sero-*K*-Set	641	89.1 (88.3–89.8)	5229/5870
All RDTs (in parallel)	829	85.9 (85.0–86.7)	5041/5870
Sero-*K*-Set + rHAT Sero-Strip (parallel)	666	88.7 (87.8–89.4)	5204/5870
HAT Sero-*K*-Set + SD Bioline (parallel)	807	86.3 (85.4–87.1)	5063/5870
rHAT Sero Strip + SD Bioline HAT (parallel)	453	92.3 (91.6–92.9)	5417/5870
All RDTs (in series)	5	99.9 (99.8–100)	5865/5870
Sero-*K*-Set + rHAT Sero-Strip (series)	17	99.7 (99.5–99.8)	5853/5870
HAT Sero-*K*-Set + SD Bioline (series)	253	95.7 (95.1–96.2)	5617/5870
rHAT Sero Strip + SD Bioline HAT (series)	8	99.9 (99.7–99.9)	5862/5870
Trypanolysis	0	100 (99.5–100)	816/816
ELISA/*T. b. gambiense*	7	99.1 (98.5–99.7)	809/816

a Denominator of 5870 for RDTs, and 816 (RDT positives only) for both trypanolysis and ELISA/*T. b. gambiense*; CI: confidence interval.

The HAT Sero-*K*-Set test showed the lowest specificity of 89.1% (88.3–89.8%), while the specificities of SD Bioline HAT and rHAT SeroStrip were 92.9% (92.2–93.5%) and 99.3% (99.0–99.5%), respectively. The highest specificity in parallel (92.3%) was observed with a combination of rHAT Sero-Strip and SD Bioline HAT. Combining RDTs in series, the highest specificity (99.9%) was observed with a combination of the three RDTs. The specificity of ELISA/*T. b. gambiense* and trypanolysis, which were performed in series on RDT positive subjects only, was 99.1% (98.5–99.7%) and 100% (99.5–100%), respectively.

## Discussion

We here describe the results of a door-to-door medical survey for HAT carried out in South–West Burkina Faso to evaluate the performance of diagnostic tests for post-elimination monitoring.

Burkina Faso, having declared only a single native case since 1993 [14], has been considered eligible for validation of elimination of HAT as a public health problem [18]. Nevertheless, the Batié health district, combines several risk factors for potential HAT transmission and re-emergence. Batié is a historical focus of sleeping sickness [16, 18] with numerous watercourses and with tsetse flies still present [11, 35]. Substantial migratory flow exists between Batié and Côte d’Ivoire, where HAT cases are still detected [30], and which has previously been the origin of imported HAT cases in Burkina Faso [26, 27, 32]. Furthermore, pigs, which are a potential animal reservoir for *T. b. gambiense* [34], are often found inside and around compounds. Moreover, it would be difficult to detect HAT cases through the current passive surveillance system [14]. Health centers are far from villages, and the closest sentinel site for HAT surveillance is 70 km away. Furthermore, no recent mass screening activities have been implemented. The Batié health district was therefore selected for this study.

Our approach combined door-to-door visits by small minimally equipped mobile teams applying RDTs only [41], with simultaneous collection of DBSs [20]. This format has already shown several advantages, first and foremost improved accessibility. The use of motorcycles allows better penetration into difficult to reach areas, and more friendly testing conditions [28, 41]. Furthermore, the exact residence of all participants was known, which facilitates parasitological follow-up if needed. As low attendance rates may be a major reason for limited effectiveness of active screening [36], and taking into account that potential existence of HAT may have been forgotten by the population and health workers [30], the team strongly invested in raising disease awareness and involved as many stakeholders as possible. These included health authorities, health workers, opinion leaders and village chiefs.

Thanks to the door-to-door format, the sensitizing efforts, and the experience of the team, almost 6000 participants could be tested within a relatively short time. However, some difficulties should be mentioned as limitations of the study. The growing artisanal gold mining sector [37] has caused demographic changes, with temporary camps, not recognized at the administrative level, close to the mining sites, while traditional villages are being deserted [2]. As a result, village population data retrieved during the planning phase were not fully reliable, while camps were not considered for the study as their population was considered too variable to allow correct parasitological follow-up of suspects. Some villages also presented a high level of insecurity and were avoided. During the screening phase, many people still refused to participate, either because they no longer knew about HAT, and/or because they were not paid for their participation. For some study participants, the blood volume collected was insufficient. During the parasitological confirmation phase, 38.4% of the RDT positive subjects could not be traced back. Loss of serological suspects for parasitological confirmation is a known drawback of serological screening with delayed laboratory tests or microscopy, even if the delay is short [41]. Furthermore, an entire village refused microscopic examination while in another village, due to an ongoing community conflict, the participation rate in parasitological confirmation was low. Poor road conditions rendered several villages inaccessible. Some serological suspects had left their village. Finally, the COVID-19 pandemic disrupted conduct of the survey. In line with the WHO guidance on Neglected Tropical Diseases (NTDs) [18], the parasitological confirmation campaign was postponed for several months. Interference of the COVID-19 pandemic with several aspects of NTD control [15] and with active screening for HAT also occurred in other countries [1]. Fortunately, among all RDT seropositives lost for confirmation, only one was positive in ELISA/*T. b. gambiense*. Since he was trypanolysis negative, was probably at low risk, and moved several hundred kilometers away, we have not continued our efforts to find him. An additional difficulty was the COVID-19 related limitations on staff time in the laboratory, which forced us to make choices. Priority was given to testing DBSs from RDT positives, as these can be considered most at risk for HAT. Furthermore, the use of molecular tests LAMP and m18S-qPCR, which are time and labor-intensive [7], was postponed.

We report for the first time the diagnostic performance of three HAT RDTs during active screening in West Africa. As no HAT cases were detected, the diagnostic sensitivity could not be assessed. For all tested RDTs, the specificities were lower than those reported for passive case detection in Côte d’Ivoire [29]. This lower specificity seems to be the most pronounced with HAT-Sero-*K*-Set, which was previously reported to be 97.8% specific (95% CI: 97.2–98.2%), while in the present study, specificity was only 89.1% (95% CI: 88.3–89.8%). In Guinea, diagnostic specificity of HAT-Sero-*K*-Set also seems to be lower in active, compared to passive, screening (B. Bucheton, personal communication). Working in open air could affect the test performance through stronger evaporation of reagents or by rendering doubtful reactions more visible due to the high light intensity. Contrary to our observations, in a study in the Democratic Republic of Congo, specificity in active screening was higher than in passive screening [31]. The specimen collection method, blood collected on heparin by venipuncture, can also be responsible for the lower specificities. In a previous test evaluation on stored plasma collected by venipuncture on heparin, relatively low specificities of around 88% were observed with HAT-Sero-*K*-Set and SD Bioline HAT [25]. Finally, an effect of environmental factors and an influence of animal trypanosomiasis might also explain our observations. No tsetse control activities have been organized in Batié in recent years and results from a previous study on the presence of anti-tsetse saliva antibodies suggest intensive human-tsetse contact [13]. Furthermore, the prevalence of animal trypanosomosis was probably close to 10%, as previously reported among bovines near the banks of the Comoé River [17]. Contact with animal trypanosomes might be responsible for false positive reactions in RDTs. This environmental context may also explain the higher RDT positivity rate in women, who spend more time in the forest for agriculture, looking for wood and sometimes for gold, with consequently increased exposure to tsetse fly bites.

As expected, the laboratory tests on DBS showed high specificity. The specificity of the ELISA/*T. b. gambiense* was similar to the specificity in Côte d’Ivoire [29], and in several studies carried out in the Democratic Republic of Congo [20, 22, 33]. The 100% specificity for trypanolysis confirms previous observations in the historical Gaoua HAT focus [12]. In this previous study, trypanolysis was carried out on plasma with *T. b. gambiense* variable antigen type LiTat 1.3 only, while in the present study, antibodies against both LiTat 1.3 and LiTat 1.5 were assessed and DBSs were used. Addition of the LiTat 1.5 variable antigen type might increase the sensitivity of trypanolysis, particularly in regions where LiTat 1.3 is not circulating [42]. Although its sensitivity is lower than on plasma, trypanolysis on DBS has been proposed as a good compromise between feasibility and sensitivity [6]. Furthermore, trypanolysis performance has been linked to prevalence levels [12]. It is therefore not surprising that the specificity of trypanolysis in Bonon in Côte d’Ivoire was lower (94.4%), as HAT cases in this focus are still observed [29].

The present study improved our knowledge of the HAT elimination status in the Batié health district. Zero parasite prevalence was observed in the 519 seropositives examined by mAECT. The zero seroprevalence in trypanolysis indicates that this population has not been exposed recently to *T. b. gambiense* [24]. Our results therefore suggest that *T. b. gambiense* no longer circulates and that zero transmission has probably been reached [5].

However, reaching this status should not be considered an end point. The risk for re-emergence in Batié persists, and continued HAT surveillance remains necessary. Furthermore, our results underline the need to train health workers regularly and to raise awareness about HAT among the population [8]. As a next step, we intend to perform a least cost analysis of different algorithm options with a projection over a 5-year time horizon. The results generated by these analyses, as well as further sensitivity estimates and further latent class analyses, will allow us to propose optimal algorithms for effective post-elimination surveillance in Burkina Faso.
